# Determining Antioxidant Activity of Cannabis Leaves Extracts from Different Varieties—Unveiling Nature’s Treasure Trove

**DOI:** 10.3390/antiox12071390

**Published:** 2023-07-06

**Authors:** Anna Stasiłowicz-Krzemień, Szymon Sip, Piotr Szulc, Judyta Cielecka-Piontek

**Affiliations:** 1 Department of Pharmacognosy and Biomaterials, Faculty of Pharmacy, Poznan University of Medical Sciences, Rokietnicka 3, 60-806 Poznan, Poland; astasilowicz@ump.edu.pl (A.S.-K.); szymonsip@ump.edu.pl (S.S.); 2 Department of Agronomy, Poznań University of Life Sciences, Dojazd 11, 60-632 Poznan, Poland; piotr.szulc@up.poznan.pl; 3 Department of Pharmacology and Phytochemistry, Institute of Natural Fibres and Medicinal Plants, Wojska Polskiego 71b, 60-630 Poznan, Poland

**Keywords:** *Cannabis sativa*, antioxidant, cannabidiol, cannabigerol, oxidative stress

## Abstract

Cannabis leaves contain a diverse range of antioxidants, including cannabinoids, flavonoids, and phenolic compounds, which offer significant health benefits. Utilising cannabis leaves as a source of antioxidants presents a cost-effective approach because they are typically discarded during the cultivation of cannabis plants for their seeds or fibres. Therefore, this presented study aimed to assess the antioxidant activity of the leaves of selected hemp cultivars, such as Białobrzeska, Tygra, and Henola, based on the results obtained with the 2,2′-Azino-bis(3-ethylbenzthiazoline-6-sulfonic acid, ferric reducing antioxidant power, cupric reducing antioxidant capacity, and 2,2-Diphenyl-1-picrylhydrazyl assays. The cannabinoid profile was analysed for the antioxidant activity to the contents of cannabidiol (CBD), cannabigerol (CBG), Δ^9^-tetrahydrocannabinol (Δ^9^-THC), and cannabichromene (CBC), determined based on chromatographic assays. The following variables were tested: the impact of various extractants (methanol, ethanol, and isopropanol), and their mixtures (50:50, *v/v*, as well as extraction methods (maceration and ultra-sound-assisted extraction) significant in obtaining hemp extracts characterised by different cannabinoid profiles. The results revealed that the selection of extractant and extraction conditions significantly influenced the active compounds’ extraction efficiency and antioxidant activity. Among the tested conditions, ultrasound-assisted extraction using methanol yielded the highest cannabinoid profile: CBD = 184.51 ± 5.61; CBG = 6.10 ± 0.21; Δ9-THC = 0.51 ± 0.01; and CBC = 0.71 ± 0.01 μg/g antioxidant potential in Białobrzeska leaf extracts.

## 1. Introduction

Cannabis has a wide range of applications, including industrial [1], ornamental [2], nutritional [3], medicinal, and recreational [4] uses. From a regulatory and application standpoint, cannabis plants are classified based on the level of Δ^9^-tetrahydrocannabinol (THC), one of the most important phytocannabinoids [5]. Plants are generally categorised and regulated as industrial hemp if they contain less than 0.3% THC in the dried flower (the specific threshold may vary by country), or as drug-type cannabis if they exceed this threshold [6]. Cannabis contains a large group of active compounds which exhibit multidirectional biological activity and can influence each other in modifying the strength and scope of pharmacological profile and mutual bioavailability [7,8,9]. The most well-known group of secondary metabolites present in cannabis are the cannabinoids. The most abundant compound is cannabidiol (CBD), which is a non-psychoactive compound found in cannabis that has shown promise in treating epilepsy, anxiety, and pain [10,11]. Tetrahydrocannabinol (THC) is the primary psychoactive secondary metabolite in cannabis and it is known for its analgesic and anti-inflammatory properties [12]. Other cannabinoids found in cannabis, such as cannabigerol (CBG), cannabichromene (CBC), and tetrahydrocannabivarin (THCV), also have potential medicinal properties [13,14]. Cannabis also contains terpenes, aromatic compounds that give the plant its distinctive smell. Terpenes have potential medicinal properties, such as anti-inflammatory, antifungal, and antibacterial effects [15]. Some terpenes found in cannabis, such as beta-caryophyllene and limonene, have been shown to have anti-inflammatory effects [16,17]. And essentially, cannabis also contains flavonoids [18]. Flavonoids are a widespread group of secondary metabolites that exhibit antioxidant activity. This activity is associated with many health-promoting effects that flavonoids might have. Among the flavonoids present in cannabis, there is a characteristic group of cannflavins. For some of the flavonoids, specific biological effects have already been defined. For example, cannflavin A, has shown potential in treating pain and inflammation [18]. Luteolin exhibits various activities, including antioxidant, anti-inflammatory, anticancer, and neuroprotective properties [19]. Quercetin demonstrates diverse activities, such as antioxidant, anti-inflammatory, antiviral, anticancer, and cardioprotective effects [20]. Currently, there is more and more discussion about the antioxidant potential of cannabinoids, especially among different cannabis varieties, which have been modified over the years to obtain selected cannabinoid profiles. As a result, they are used as an industrial material, and their leaves are post-production waste. Numerous studies have investigated the antioxidant activity of the compounds found in hemp. For example, a study by Tura et al. found that CBD had significant antioxidant activity in vitro [21]. Another study investigated the antioxidant activity of CBC, CBG, and other compounds found in hemp [22].

Additionally, many studies investigated the effects of CBD on oxidative stress [23,24,25,26,27,28]. The researchers found that CBD could reduce oxidative stress and inflammation in cell cultures and in animal models. The researchers found that CBD had significant antioxidant and anti-inflammatory effects and the potential to treat neurodegenerative diseases [29]. Various methods have been used to analyse the antioxidant activity of hemp compounds, including the oxygen radical absorbance capacity (ORAC) assay, the 2,2-diphenyl-1-picrylhydrazyl (DPPH) assay, and the ferric reducing antioxidant power (FRAP) assay [30]. These assays measure the ability of a compound to scavenge free radicals or reduce oxidised compounds. Overall, the literature suggests that the compounds found in hemp, including CBD, CBC, CBG, and other flavonoids and phenolic compounds, have significant antioxidant activity. This activity may help protect against oxidative stress and inflammation in the body and may have potential in the treatment and prevention of various diseases. Nevertheless, additional research is needed to attain a comprehensive understanding of the mechanisms of action and potential therapeutic applications of these compounds found in cannabis leaves.

While hemp leaves are often considered waste products in the hemp industry, they are typically discarded after extracting the desired compounds from the flowers and buds. However, recent studies have shown that hemp leaves also contain high levels of active compounds, including cannabinoids, terpenes, and flavonoids, which could be utilised for various applications [31]. In addition to their high concentration of active compounds, hemp leaves could provide a zero-waste approach to the hemp industry. Instead of being discarded, the leaves could be used to extract active compounds, providing an additional source of revenue for hemp growers and processors.

Furthermore, using hemp leaves for extraction could also contribute to the sustainable development of the hemp industry. The industry can reduce waste and minimise its environmental impact by utilising all hemp plant parts, including the leaves. However, extracting active compounds from hemp leaves may require different methods and solvents than extracting from flowers and buds. For example, a recent study showed that ethanol was the most effective solvent for extracting cannabinoids from hemp leaves, while other solvents, such as hexane and chloroform, had lower extraction efficiencies. Therefore, further research is needed to optimise the extraction of active compounds from hemp leaves and to determine their potential applications. Nevertheless, using hemp leaves as a source of active compounds could provide a promising avenue for developing a sustainable and economically viable hemp industry.

Several factors, such as the type and amount of solvent, temperature, and extraction time, often influence the efficiency of the extraction process [32,33]. Different solvents have varying affinities for specific compounds, leading to their yields and purity variations. Therefore, selecting an appropriate solvent is critical in achieving the desired yield and quality of the extracted compounds. Alcohols, such as ethanol and methanol, are commonly used solvents in hemp extraction due to their high solubility in both water and nonpolar solvents. In addition to the type of alcohol used, other factors, such as extraction time and temperature, can also affect the yield and quality of the extracted compounds. Shorter extraction times and lower temperatures can preserve the quality of the extracted compounds, while longer extraction times and higher temperatures can lead to degradation and loss of potency.

Utilising cannabis leaves as antioxidants holds a novel potential in disease prevention [34]. Despite being often disregarded as waste during cultivation, cannabis leaves possess abundant antioxidants, including cannabinoids. Identifying the cannabis variety with the highest antioxidant potential is crucial for optimisation. Cultivating the variety currently grown on a large scale with superior antioxidant properties would ensure a steady supply of antioxidant-rich cannabis leaves available for many people from different parts of the world, promoting sustainability, and minimising waste. Expanded cultivation efforts could also facilitate genetic advancements, enhancing the antioxidant properties and overall effectiveness of cannabis leaves as antioxidants.

This novel approach of focusing on hemp leaves was implemented in this work. The study investigated the impact of different extractants and their mixtures, including methanol, ethanol, and isopropanol, on the extraction efficiency of active compounds from three varieties of hemp leaves, namely Białobrzeska, Tygra, and Henola. The researchers determined the content of cannabinoids and assessed the antioxidant activity using several models, including 2′-Azino-bis(3-ethylbenzthiazoline-6-sulfonic acid (ABTS), cupric reducing antioxidant capacity (CUPRAC), FRAP, and DPPH assays.

## 2. Materials and Methods

### 2.1. Materials

The plant material for studies—Białobrzeskie, Tygra, and Henola varieties—were donated from the Experimental Station for the Cultivar Testing in Chrząstowo, belonging to the Research Centre for Cultivar Testing in Słupia Wielka. The forecrop for hemp in 2022 was sugar beet. Individual tillage operations were carried out in accordance with agrotechnical recommendations for this species (winter plowing 29 October 2021; 17 March 2022 harrow + spear, 6 May 2022 cultivation unit; 9 May 2022 sowing). The day after the sowing of hemp (10 May 2022), Boxer 800 EC (Syngenta, Warsaw, Poland) herbicide was applied at a rate of 2.6 L/ha. Mineral fertilisation was carried out based on the following mineral fertilisers: Lubofos 12 (200 kg/ha), potassium salt (183 kg/ha), enriched superphosphate (115 kg/ha), urea (159 kg/ha), and salmag (119 kg/ha). The soil of the experimental field was classified as IIIa, complex 2. The analysed soil’s top horizons are classified as loamy sands, with a clay fraction content of 4%, dust fraction of 14%, and sand fraction of 83% in terms of grain size. The eluvial level exhibits a slightly lower proportion of clay fraction and dust fraction. The enrichment (B) and bedrock levels demonstrate higher compactness. The pH, as determined through aqueous extract measurement, was found to be 6.80, while in KCl, the pH value was slightly lower by approximately 0.5 units, aligning with the upper range of slightly acidic values. Furthermore, the organic carbon content registered around 1%, equivalent to a humus content of 1.7%. The total nitrogen content measured approximately 0.086%, resulting in a C:N ratio of approximately 12:1. Moreover, the favourable thermal and moisture conditions experienced throughout the growing season greatly contributed to the robust growth and development of cannabis plants. Standards of four cannabinoids, namely, cannabidiol, delta-9-tetrahydrocannabinol, cannabigerol, and cannabichromene, were obtained from the Sigma-Aldrich (Poznan, Poland).

Trifluoric acid and acetonitrile (high-performance liquid chromatography [HPLC] grade) were provided by Merck (Darmstadt, Germany). High-quality pure water was prepared using a Direct-Q 3 UV purification system (Millipore, Molsheim, France; model Exil SA 67120). 2,2-Diphenyl-1-picrylhydrazyl, iron (III) chloride hexahydrate, 2,2′-azino-bis(3-ethylbenzothiazoline-6-sulfonic acid), neocuproine, 2,4,6-Tri(2-pyridyl)-s-triazine, and trolox were supplied by Sigma-Aldrich (Schnelldorf, Germany). Sodium chloride, and sodium hydrogen phosphate, were purchased from Avantor Performance Materials (Gliwice, Poland). Ammonium acetate (NH4Ac) and methanol were obtained from Chempur (Piekary Śląskie, Poland). Cupric chloride dihydrate, ethanol (96%), isopropanol (99%), acetic acid (99.5%), and sodium acetate trihydrate were obtained from POCH (Gliwice, Poland).

### 2.2. Extraction

Bialobrzeskie, Tygra, and Henola leaves were grounded, dried, and placed in flasks (1.0 g). The flasks were filled with 50.0 mL of solvent/solvent mixture (methanol/ethanol/isopropanol/50:50 (*v*/*v*) methanol:ethanol/50:50 (*v*/*v*) ethanol:isopropanol/50:50 (*v*/*v*) methanol:isopropanol). Two methods of extraction were implemented: maceration for 24 h at room temperature in shaking with the rotation frequency of 300 rpm (PSU 10i shaker, Biosan, UK), and ultrasound-assisted extraction for 1 h at 40 °C (constant, uninterrupted sonication, frequency 37 kHz, ultrasonic peak max. 800 W) (Thermo Fisher Scientific, Waltham, MA, USA). The extracts were filtered and supplemented to a concentration of 20 mg/mL.

### 2.3. Chromatographic Analysis

**Figure 1:**
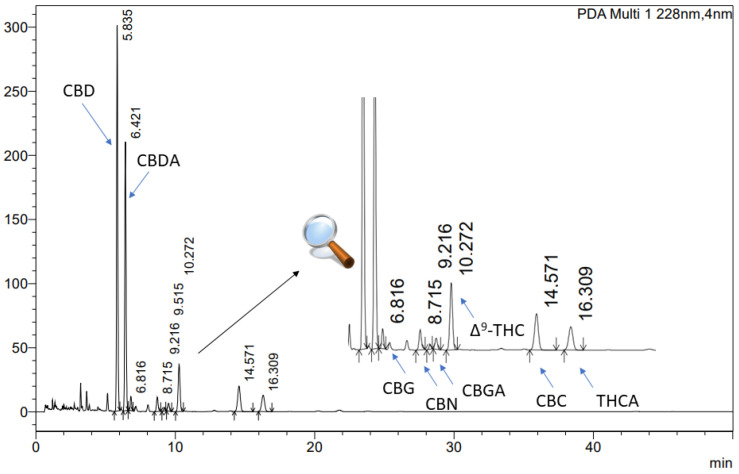
The chromatogram of cannabinoids in *Cannabis inflorescences* extracts: CBD (cannabidiol), CBG (cannabigerol), Δ^9^-THC ((-)-delta 9-tetrahydrocannabinol), and CBC (cannabichromene).

To analyse the cannabinoid profile of the extracts, an ultra-high-performance liquid chromatography technique with a diode array detector (UHPLC-DAD) was employed. The study was conducted using a CORTECS Shield RP18, 2.7 µm; 150 mm × 4.6 mm as a stationary phase, with a mobile phase of 0.1% trifluoric acid and acetonitrile (41:59, *v*/*v*). The flow rate was maintained at 2.0 mL/min, and the column temperature was set to 35 °C. The analysis time was 50 min, with an injection volume of 10.0 µL and a detection wavelength of 228 nm. The retention times of cannabinoids were as followed: CBD approx. 5.835 min, CBG approx. 6.816 min, Δ^9^-THC 10.272 approx. min, and CBC 14.571 approx. min (Figure 1). The data were processed using LabSolutions LC software (version 1.86 SP2) provided by Shimadzu Corp., Kyoto, Japan.

### 2.4. Antioxidant Activity

Four different assays, namely, 2,2-Diphenyl-1-picrylhydrazyl (DPPH), 2,2′-Azino-bis(3-ethylbenzthiazoline-6-sulfonic acid (ABTS), cupric reducing antioxidant capacity (CUPRAC), and ferric reducing antioxidant power (FRAP) assays, were used to determine the antioxidant activity of the extracts. The screening of the extracts’ antioxidant activity was carried out prior to each assay, by testing the extracts at decreasing concentrations. The antioxidant activity of Trolox was also measured at an appropriate concentration range for inhibiting radicals in the DPPH and ABTS assays or performing redox reactions in the CUPRAC and FRAP assays. A linear regression equation was constructed between the concentration of Trolox and its scavenging percentage or absorbance, depending on the assay used. The results were expressed as mg Trolox per gram of plant material, based on the antioxidant properties of the extracts in all four assays [35,36].

DPPH assay was carried out in a 96-well plate, the samples were measured using spectrophotometry [37]. The primary reagent consisted of a 0.2 mM methanol solution of DPPH. To initiate the reaction, 25.0 µL of the extracts/trolox solution was pipetted to 175.0 µL of the DPPH solution, and the plate was incubated in darkness at room temperature for 30 min while shaking. The absorbances were obtained at 517 nm on a plate reader (Multiskan GO, Thermo Fisher Scientific, Waltham, MA, USA). The absorbance of the blank (mixture of DPPH solution and solvent) was also measured at 517 nm. Each sample was evaluated for its own absorbance at 517 nm. The percentage of inhibition of DPPH radicals by the extracts/trolox was determined using the provided formula.

(1) $$DPPH\;scavenging\;activity\;(\%)\frac{A_{o\;}-A_{i}}{A_{o}}\times 100\%$$

in the formula, *A_o_* is the control sample absorbance, whilst *A_i_* is the test sample absorbance. Each measurement was performed six times.

Another assay used to evaluate the radical scavenging potential of the samples was the ABTS assay [38]. During the study, the generation of green cation radicals occurs as a result of electron loss from the nitrogen atoms of ABTS through the action of potassium persulfate. The antioxidant converts the green ABTS radical into a colourless neutral form. To perform the ABTS assay, 200.0 μL of ABTS^•+^ solution and 10.0 μL of the extract/trolox solution were pipetted to 96-well plates and incubated for 10 min in darkness at room temperature with shaking [39]. Following the incubation period, the absorbance values were recorded at a wavelength (λ) of 734 nm. using a Multiskan GO plate reader (Thermo Fisher Scientific, Waltham, MA, USA). The absorbance values were also determined for the control mixture of solvent and ABTS, and for the wells filled with extract and water (extracts’ absorbance) at 734 nm. The inhibition of ABTS^•+^ by the extracts and Trolox was determined with the use of the provided formula:

(2) $$\mathrm{ABTS}\;\mathrm{scavenging}\;\mathrm{activity}\;(\%)=\frac{A_{0}-A_{1}}{A_{0}}\times 100\%$$

where:

*A*_0_—the absorbance of the control;

*A*_1_—the absorbance of the sample.

To evaluate the reducing potential of extracts, the CUPRAC and FRAP assays were used. The CUPRAC method involved the oxidation of phenolic groups of antioxidants to quinones, leading to the reduction of the neocuproine and copper (II) ion complex to the neocuproine and copper (I) ion complex, resulting in a bluish to yellow colour change [40]. An amount of 50.0 µL of the extract/trolox solution and 150.0 µL of the CUPRAC reagent were pipetted to the plate and incubated for 30 min at room temperature in darkness [39]. Then, the absorbance was measured at 450 nm using a plate reader (Multiskan GO, Thermo Fisher Scientific, Waltham, MA, USA). The control and extracts’ own absorbance were also studied.

The FRAP technique, involved the reduction of colourless Fe^3+^ ions to Fe^2+^ ions, leading to the formation of a dark blue complex with 2,4,6-tris(2-pyridyl)-1,3,5-triazine (TPTZ) [39]. This reaction was monitored by measuring the absorbance of the solution at 593 nm using a plate reader (Multiskan GO, Thermo Fisher Scientific, Waltham, MA, USA) after incubating the mixture of 25.0 µL of the extract/trolox solution and 175.0 µL of FRAP mixture (25 mL acetate buffer, 2.5 mL TPTZ solution, and 2.5 mL of FeCl_3_·6H_2_O solution) in the plate for 30 min at 37 °C in dark conditions. Control and extracts’ absorbance were also studied, and the measurements were performed in six replicates.

### 2.5. Analysis of the Results

To conduct the statistical analysis, Statistica 13.3 software (StatSoft Poland, Krakow, Poland) was used. Data are presented as mean values ± standard deviations (SD). Experimental data of the antioxidant properties of cannabis leaves extracts were analysed using the skewness and kurtosis tests to determine the normality of each distribution, and the equality of variances was studied with the Levene’s test. Statistical significance was determined with the use of a one-way analysis of variance (ANOVA) followed by the Bonferroni post hoc test (for comparison of the experimental results for each cannabis leaves extract). Differences were considered significant at *p* < 0.05. PCA, which was used to explain and interpret interdependence between the cannabinoids’ profile and their impact on the antioxidant activity of the cannabis leaves extracts was conducted using PQStat v. 1.8.4.140 software (Poznań, Poland). To determine the validity of using the PCA, the Bartlett Test and the Kaiser–Mayer–Olkin coefficient were studied. The principal components were based on the correlation matrix.

To determine the cannabis leaves extract with the strongest antioxidant activity (DPPH, ABTS, CUPRAC, FRAP methods), a multidimensional comparative analysis (MCA), which compares multi-feature objects, was performed [41,42]. Synthetic indicators are the main criterion for organising the examined results and their ranking with the use of multidimensional comparative analysis. In the process of normalisation, the considered diagnostic features have been assigned an equal meaning for the assessment of objects. Standardisation was used for the normalisation of variables. Synthetic measures were calculated, and rankings of regions were prepared.

## 3. Results

Cannabis is a rich source of secondary plant metabolites, including cannabinoids, terpenes, and flavonoids, which contribute to its unique aroma, taste, and therapeutic properties. Cannabinoids are the most well-known secondary metabolites in cannabis, with over 100 cannabinoids identified [43]. These cannabinoids interact with the endocannabinoid system in the human body to produce various physiological effects. Overall, the secondary metabolites in cannabis are believed to work synergistically to produce the therapeutic effects associated with cannabis use. Understanding the composition of these secondary metabolites in different cannabis strains is important for optimising their therapeutic potential. In this study, the content of four cannabinoids, namely, CBD, CBG, Δ^9^-THC, and CBC, were determined with the UHPLC-DAD method. The results are presented in Table 1.

The highest content of CBD was noted in Henola leaves extract obtained by maceration with methanol, 210.91 ± 3.14 (μg/g), while the highest CBG content was found in Tygra leaves extract obtained by maceration with methanol and ethanol—8.67 ± 0.61 (μg/g), 8.64 ± 0.55 (μg/g), respectively. The most potent in Δ^9^-THC was Białobrzeskie leaves extract obtained by ultrasound-assisted extraction with methanol 0.51 ± 0.01 (μg/g), while Tygra leaves extract obtained by ultrasound-assisted extraction with isopropanol had the highest content of CBC—0.85 ± 0.03 (μg/g).

It is known that antioxidants can neutralise free radicals, which are highly reactive molecules that can damage cells, DNA, and other biomolecules [34,44,45,46]. Antioxidants can prevent or reduce oxidative stress by neutralising free radicals before they can cause damage.

**Figure 2:**
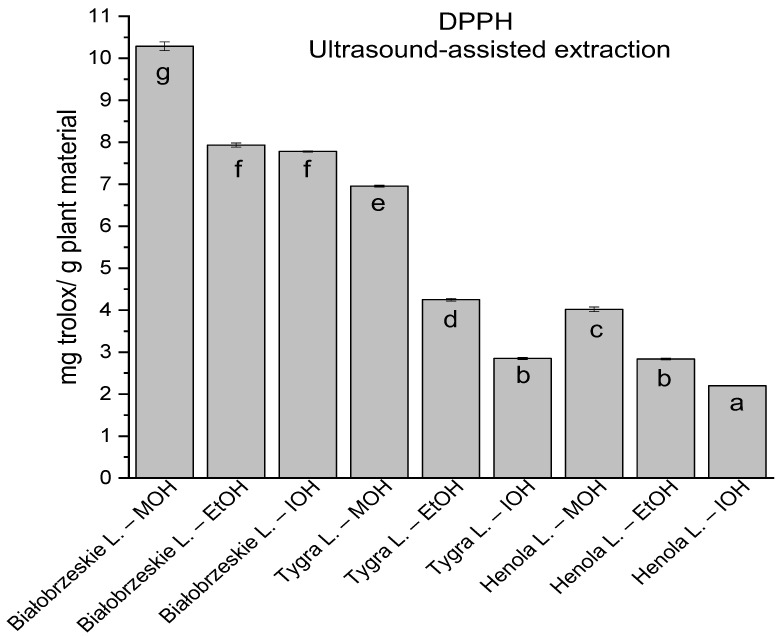
The results of the antioxidant activity of Białobrzeskie, Henola, and Tygra leaves extracts obtained by ultrasound-assisted extraction (U-A.E) with the use of methanol (MOH), ethanol (EtOH), isopropanol (IOH), presented as mg trolox/g plant material studied in DPPH assay. Columns with different superscript letters (a–g) differ significantly (*p* < 0.05).

**Figure 3:**
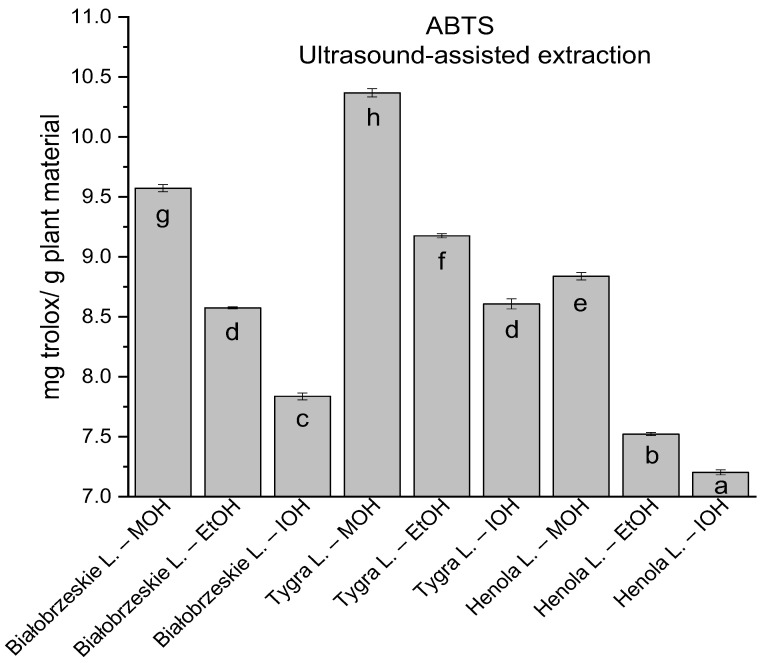
The results of the antioxidant activity of Białobrzeskie, Henola, and Tygra leaves extracts obtained by ultrasound-assisted extraction (U-A.E) with the use of methanol (MOH), ethanol (EtOH), isopropanol (IOH), presented as mg trolox/g plant material studied in ABTS assay. Columns with different superscript letters (a–h) differ significantly (*p* < 0.05).

**Figure 4:**
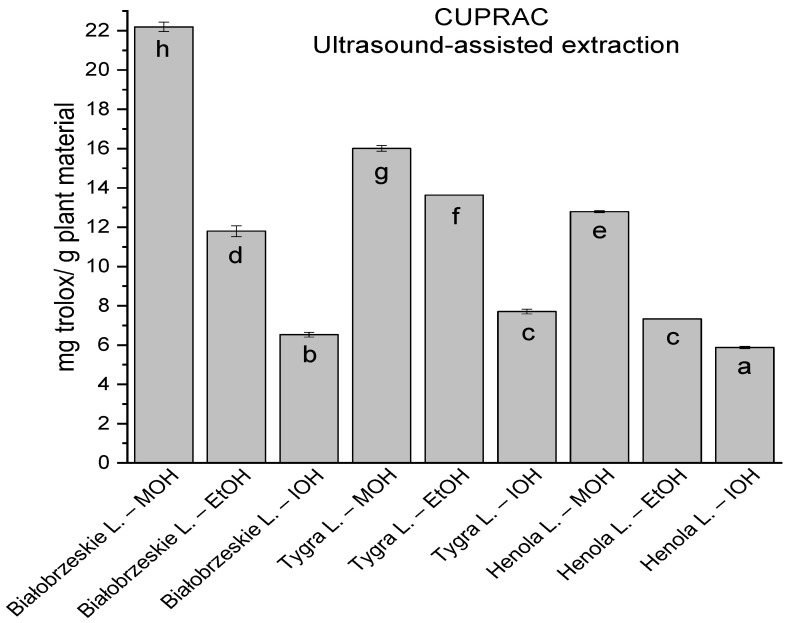
The results of the antioxidant activity of Białobrzeskie, Henola, and Tygra leaves extracts obtained by ultrasound-assisted extraction (U-A.E) with the use of methanol (MOH), ethanol (EtOH), isopropanol (IOH), presented as mg trolox/g plant material studied in CUPRAC assay. Columns with different superscript letters (a–h) differ significantly (*p* < 0.05).

**Figure 5:**
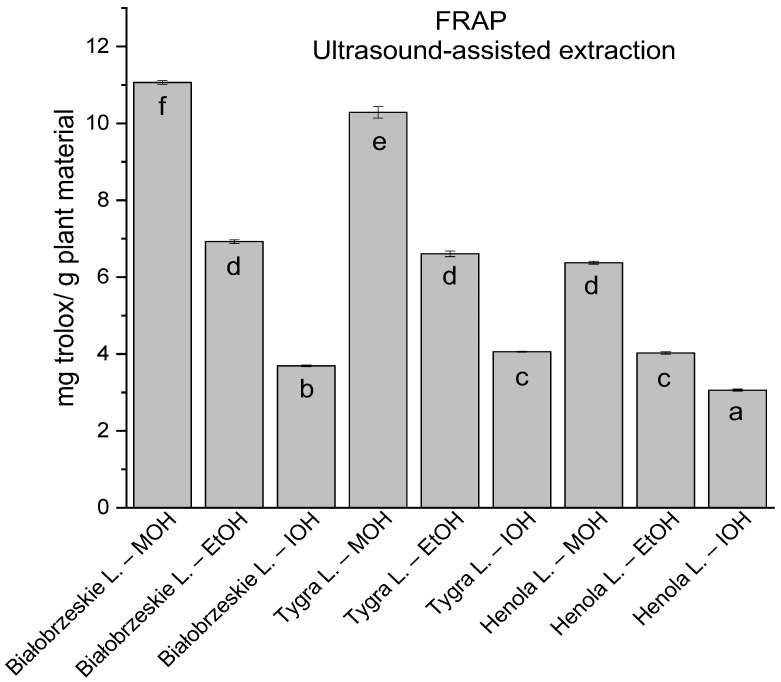
The results of the antioxidant activity of Białobrzeskie, Henola, and Tygra leaves extracts obtained by ultrasound-assisted extraction (U-A.E) with the use of methanol (MOH), ethanol (EtOH), isopropanol (IOH), presented as mg trolox/g plant material studied in FRAP assay. Columns with different superscript letters (a–f) differ significantly (*p* < 0.05).

In the group of extracts obtained by ultrasound-assisted extraction with single-component extractant (MOH/EtOH/IOH) (Figure 2, Figure 3, Figure 4 and Figure 5; Table S3, Supplementary Materials), the greatest results in DPPH, CUPRAC, and FRAP assays were obtained mainly by Białobrzeskie leaves extract obtained with MOH—10.288 ± 0.103 mg trolox/g plant material, 22.195 ± 0.242 mg trolox/g plant material, and 11.066 ± 0.048 mg trolox/g plant material, respectively. In the ABTS study, the most significant antioxidant potential was noted for Tygra leaves extract obtained with MOH 10.368 ± 0.035 103 mg trolox/g plant material. Summarily, the multidimensional comparative analysis (MCA) allowed to emerge as the most potent antioxidant from this group the Białobrzeskie leaves extract obtained with MOH.

**Figure 6:**
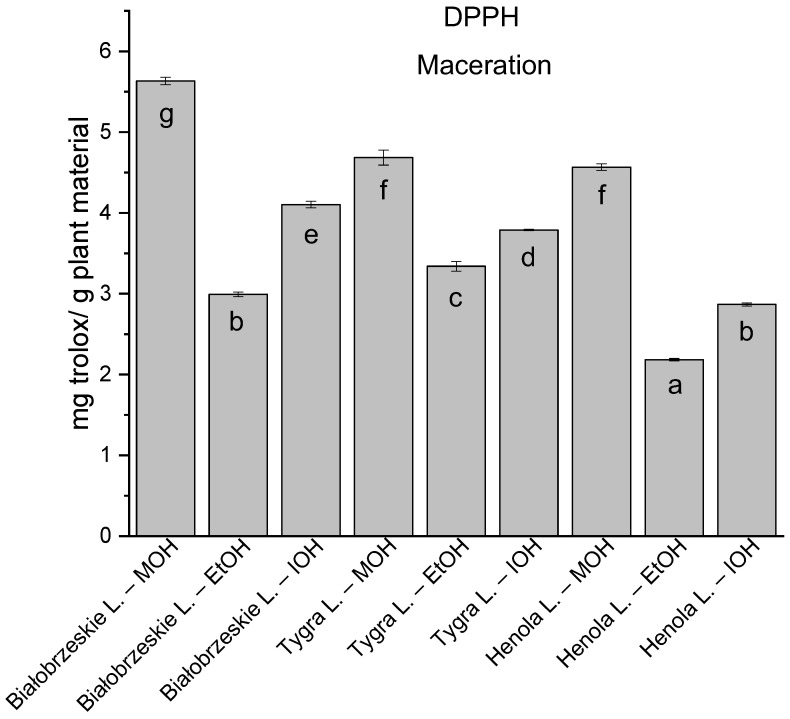
The results of the antioxidant activity of Białobrzeskie, Henola, and Tygra leaves extracts obtained by maceration with the use of methanol (MOH), ethanol (EtOH), isopropanol (IOH), presented as mg trolox/g plant material studied in DPPH assay. Columns with different superscript letters (a–g) differ significantly (*p* < 0.05).

**Figure 7:**
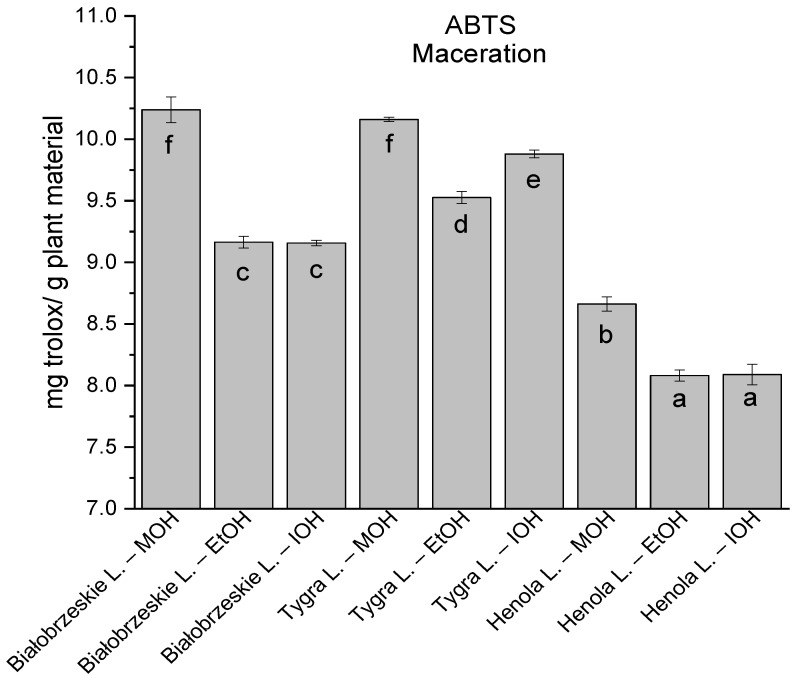
The results of the antioxidant activity of Białobrzeskie, Henola, and Tygra leaves extracts obtained by maceration with the use of methanol (MOH), ethanol (EtOH), isopropanol (IOH), presented as mg trolox/g plant material studied in ABTS assay. Columns with different superscript letters (a–f) differ significantly (*p* < 0.05).

**Figure 8:**
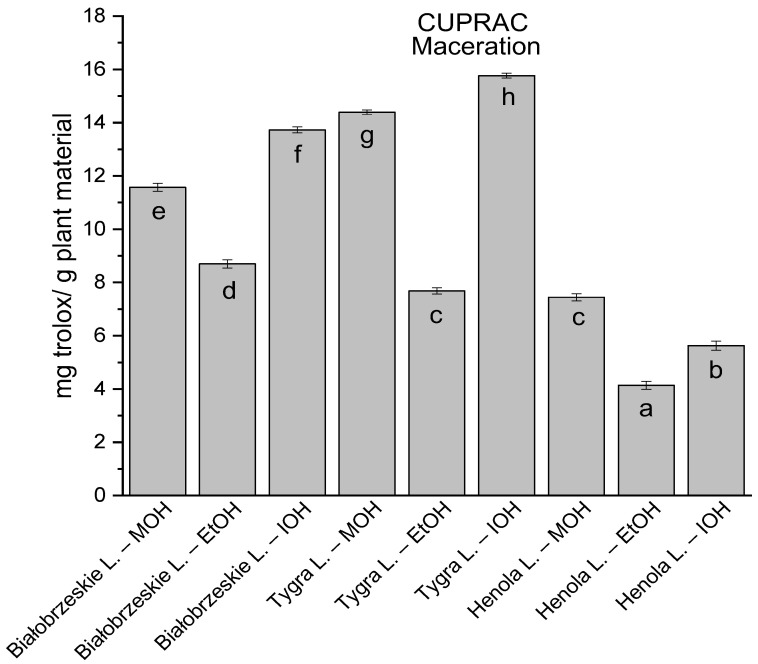
The results of the antioxidant activity of Białobrzeskie, Henola, and Tygra leaves extracts obtained by maceration with the use of methanol (MOH), ethanol (EtOH), isopropanol (IOH), presented as mg trolox/g plant material studied in CUPRAC assay. Columns with different superscript letters (a–h) differ significantly (*p* < 0.05).

**Figure 9:**
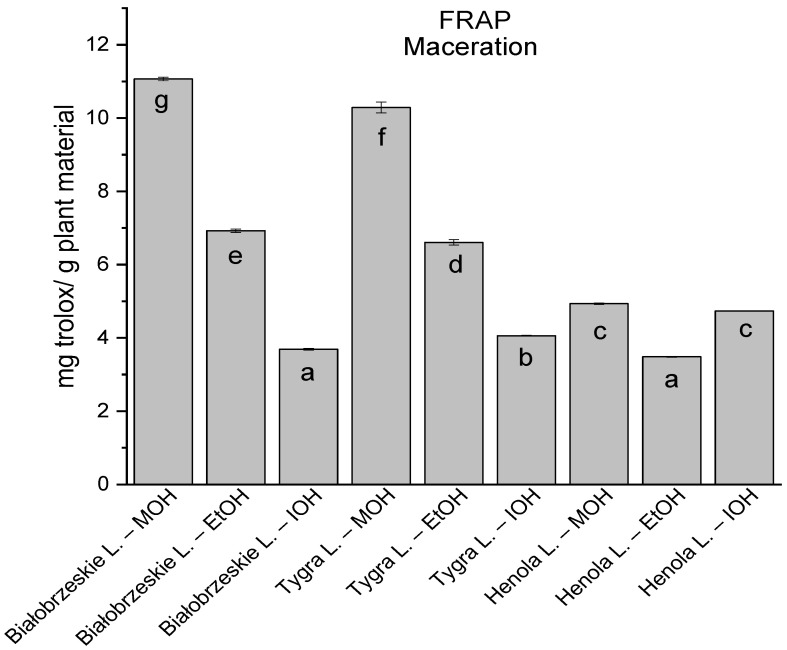
The results of the antioxidant activity of Białobrzeskie, Henola, and Tygra leaves extracts obtained by maceration with the use of methanol (MOH), ethanol (EtOH), isopropanol (IOH), presented as mg trolox/g plant material studied in FRAP assay. Columns with different superscript letters (a–g) differ significantly (*p* < 0.05).

Among macerated extracts with MOH/EtOH/IOH (Figure 6, Figure 7, Figure 8 and Figure 9; Table S2, Supplementary Materials), methanol Białobrzeskie leaves extract showed the greatest antioxidant potential in assays: DPPH—5.632 ± 0.046 mg trolox/g plant material, ABTS 10.239 ± 0.105 mg trolox/g plant material and FRAP 11.066 ± 0.048 mg trolox/g plant material. In the CUPRAC assay, the most potent was Tygra leaves extract macerated by isopropanol 15.766 ± 0.091 mg trolox/g plant material. The MCA has identified methanol Tygra leaves extract as the one with the strongest antioxidant potential within this group; however, methanol Białobrzeskie leaves extract was very close in the ranking, taking the second place in this group.

**Figure 10:**
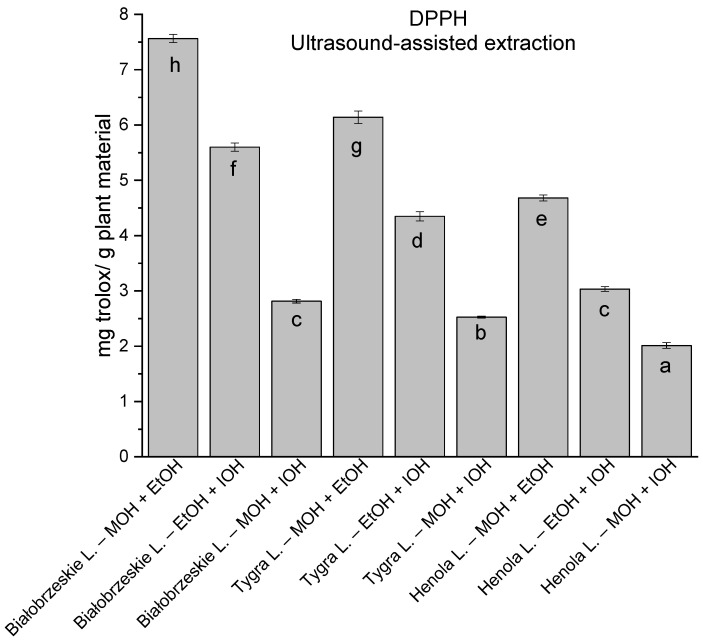
The results of the antioxidant activity of Białobrzeskie, Henola, and Tygra leaves extracts obtained by ultrasound-assisted extraction (U-A.E) with the use of 50:50 (*v*/*v*) mixtures of methanol (MOH), ethanol (EtOH), and isopropanol (IOH), presented as mg trolox/g plant material studied in DPPH assay. Columns with different superscript letters (a–h) differ significantly (*p* < 0.05).

**Figure 11:**
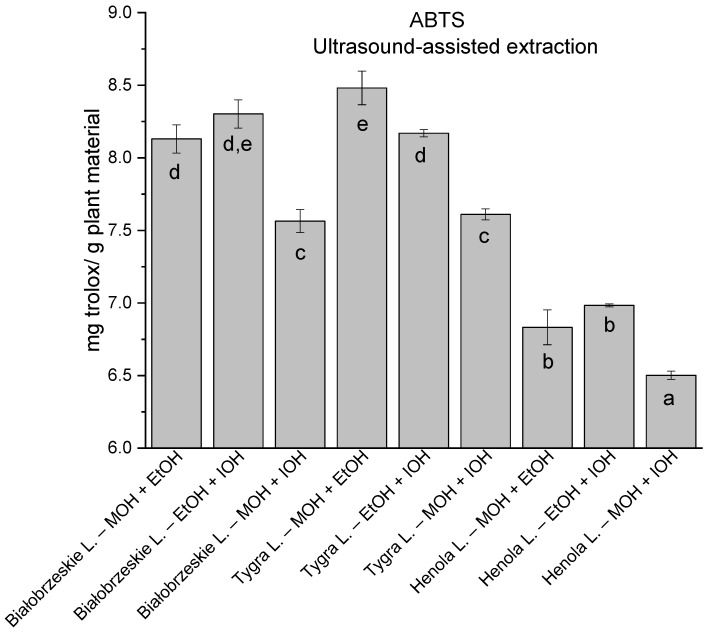
The results of the antioxidant activity of Białobrzeskie, Henola, and Tygra leaves extracts obtained by ultrasound-assisted extraction (U-A.E) with the use of 50:50 (*v*/*v*) mixtures of methanol (MOH), ethanol (EtOH), and isopropanol (IOH), presented as mg trolox/g plant material studied in ABTS assay. Columns with different superscript letters (a–e differ significantly (*p* < 0.05).

**Figure 12:**
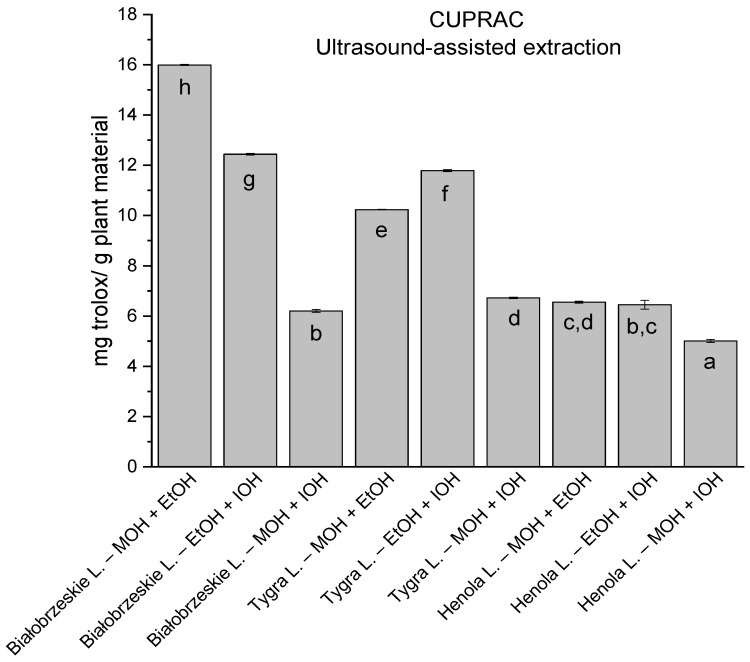
The results of the antioxidant activity of Białobrzeskie, Henola, and Tygra leaves extracts obtained by ultrasound-assisted extraction (U-A.E) with the use of 50:50 (*v*/*v*) mixtures of methanol (MOH), ethanol (EtOH), and isopropanol (IOH), presented as mg trolox/g plant material studied in CUPRAC assay. Columns with different superscript letters (a–h) differ significantly (*p* < 0.05).

**Figure 13:**
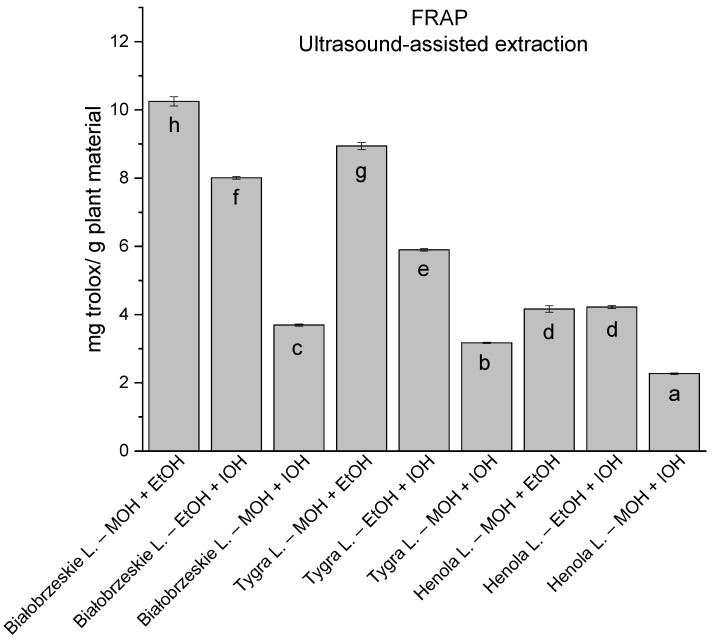
The results of the antioxidant activity of Białobrzeskie, Henola, and Tygra leaves extracts obtained by ultrasound-assisted extraction (U-A.E) with the use of 50:50 (*v*/v) mixtures of methanol (MOH), ethanol (EtOH), and isopropanol (IOH), presented as mg trolox/g plant material studied in FRAP assay. Columns with different superscript letters (a–h) differ significantly (*p* < 0.05).

In the group of extracts prepared by ultrasound-assisted extraction with the use of two-component extractants (Figure 10, Figure 11, Figure 12 and Figure 13; Table S3, Supplementary Materials), in DPPH, CUPRAC, and FRAP assays, one extract—Białobrzeskie leaves extract—obtained with methanol and ethanol (50:50, *v*/*v*) is distinguished with results 7.563 ± 0.075 mg trolox/g plant material, 15.992 ± 0.024 mg trolox/g plant material, 10.250 ± 0.135 mg trolox/g plant material, respectively. However, in ABTS the most antioxidant potential was assessed to Tygra leaves extract obtained also with methanol and ethanol (50:50, *v*/*v*)—8.481 ± 0.116 mg trolox/g plant material. Within this group, MCA provides Białobrzeskie leaves extract obtained with methanol and ethanol (50:50, *v*/*v*) as the strongest antioxidant.

MCA was also performed to compare all of the extracts and to choose the strongest one, the Bialobrzeskie leaves extract obtained with ultrasound-assisted extraction with MOH was determined as the most potent in this direction of biological activity.

**Figure 14:**
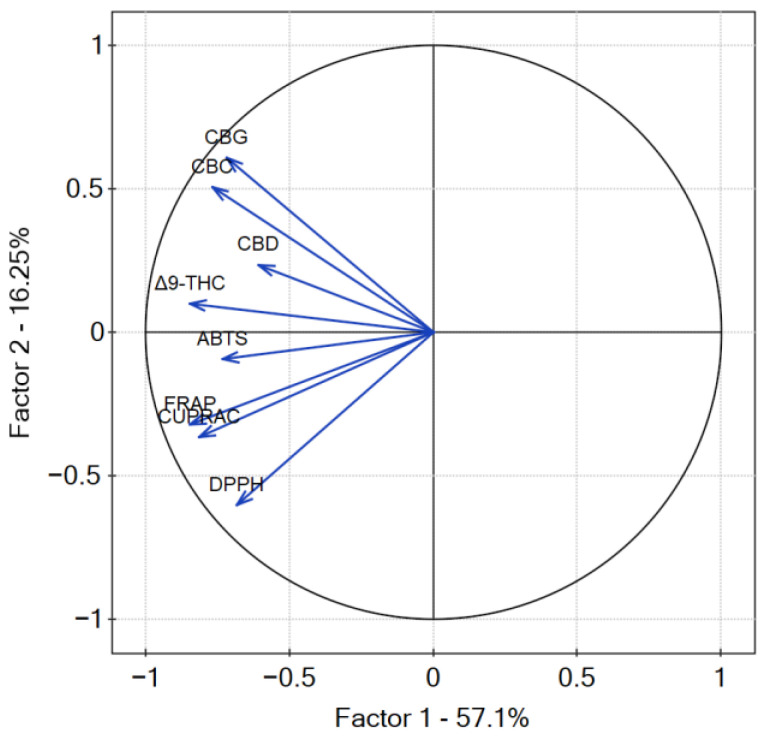
Contributions of variables (DPPH, ABTS, CUPRAC, FRAP, cannabinoids content) to PCs.

The *p*-value of Bartlett’s statistics indicates a significant correlation of variables. The obtained Kaiser–Mayer–Olkin coefficient was middling—0.69—and thus, PCA was performed. The findings from the PCA analysis provide valuable insights into the relationship between the measured variables, namely, the content of cannabinoids and the antioxidant potential of cannabis leaves extracts. PCA analysis revealed that the percentage of variation in the samples explained by factor 1 (principal component PC1) was 57.1% and 16.25 by factor 2 (principal component PC2) (Figure 14). The dominance of PC1 in explaining the variation suggests that it captures the major sources of diversity among the samples. All of the studied results showed a negative input to factor 1, whilst PC2 was negatively correlated with antioxidant results and positively correlated with the content of cannabinoids. The negative correlation of PC2 with antioxidant results and its positive correlation with cannabinoid content highlights the potential interplay between these factors in shaping the observed outcomes. The higher values of the coordinates of the vector end are noticed for PC1 than for PC2. The highest correlation between cannabinoid content and their antioxidant activity was noted for Δ^9^-THC and CBD according to the smallest angle between the vectors. CBC and CBG content showed less correlation with the antioxidant activity of cannabis leaves extracts antioxidant activity, as the angle between the vectors is greater than for Δ^9^-THC and CBD. However, it is well known that there is an intra–entourage interaction between cannabinoids in the human body [47]. This cooperative relationship enhances the overall therapeutic potential and effectiveness of cannabis-based treatments.

## 4. Discussion

Cannabinoids exhibit a range of biological effects, including analgesic, anti-inflammatory, neuroprotective, immunomodulatory, and anti-tumour properties, underscoring their multifunctional roles in diverse physiological processes. Cannabinoids also play a role as antioxidants. The relationship between the cannabinoid profile and antioxidant activity refers to their antioxidant potential. Cannabinoids, such as cannabidiol (CBD) and tetrahydrocannabinol (THC), have gained significant attention for their diverse therapeutic potential, including their antioxidant effects. In the study of Atalay et al., it was summarised that CBD exhibits potent antioxidant activity and can protect cells from induced oxidative stress [48]. In addition, CBD is able to reduce inflammation by reducing the production of pro-inflammatory cytokines, for instance, tumour necrosis factor-alpha (TNF-α) and interleukin-1 beta (IL-1β). These effects are mediated by the activation of the nuclear factor erythroid 2-related factor 2 (Nrf2) pathway. The antioxidant potential of cannabinoids, such as CBD, goes beyond their molecular structure, as they have been shown to upregulate the expression of endogenous antioxidant systems, including superoxide dismutase (SOD) and also glutathione peroxidase (GPx), through the activation of the Nrf2/Keap1 nuclear complex [49]. In another study, CBD treatment reduced the extent of liver damage caused by I/R injury, as evidenced by decreased levels of serum markers of liver injury and reduced histopathological changes in liver tissue [50]. CBD treatment also reduced oxidative stress in the liver, which is evidenced by the observed reduction in malondialdehyde levels and concurrent enhancement in the activity of antioxidant enzymes, such as superoxide dismutase and catalase. CBD treatment reduced cell death in the liver, as evidenced by reduced levels of apoptotic markers. In di Giacomo et al. study [51], CBD, and CBG antioxidant potential were studied in rat astrocytes and isolated cortexes. Both cannabinoids significantly increased cell viability and decreased the levels of ROS in astrocytes exposed to hydrogen peroxide-induced oxidative stress. Moreover, CBD and CBG significantly reduced lipid peroxidation and increased the activity of antioxidant enzymes in astrocytes. In another study, Kubiliene et al. [52], investigated the effect of cannabis sativa L. extract on oxidative stress markers in vivo. The administration of cannabis extract reduced markers of oxidative stress, such as malondialdehyde (MDA), and protein carbonyl (PC) in the liver and kidney tissues of rats. Moreover, the extract increased the activity of enzymes, superoxide dismutase (SOD), and catalase (CAT) in both liver and kidney tissues. The extract also increased the total antioxidant capacity (TAC) in the plasma of rats. The cannabis plant is known to contain a wide variety of secondary metabolites such as cannabinoids, terpenes, and flavonoids, which contribute to its pharmacological effects. For the antioxidant potential of the cannabis plant not only cannabinoids might be responsible but also terpenes [53,54], or flavonoids [55,56]. In a study on different varieties, the antioxidant potential was also changeable [57], confirming the thesis. The composition and quantity of these compounds can vary significantly among different cannabis varieties, making the cannabis market rich in diversity. Therefore, it is important for consumers to be aware of the differences in cannabis plant varieties, and to select cannabis products that are appropriate for their specific needs. The differences are noticeable in various biological activity directions, also in antioxidant potential, which was proven within this study.

Another aspect highlighted in this study is that not only cannabis flowers are rich in cannabinoids, but leaves are also a valuable source of cannabinoids and other health-promoting secondary plant metabolites. The study highlights the importance of exploring the potential of underutilised cannabis leaves, which show promise in the prevention of diseases. By exploring the utilisation of cannabis leaves, rather than solely relying on inflorescence, opportunities for therapeutic applications can be expanded, contributing to advancements in medical and supplementary industries. Other studies in this field confirm this approach. The study of Jin et al. [58] showed that inflorescences and leaves are relatively abundant in cannabinoids, monoterpenoids, sesquiterpenoids, and flavonoids, while stem barks and roots contain triterpenoids and sterols. In recent research conducted on the Industrial Hemp Futura 75 Cultivar [59], the phytochemical profile of the leaves was analysed to explore their potential health-promoting compounds. The study revealed the presence of various compounds, including a newly identified cannabinoid derivative, and seven known components, such as CBD, CBDA, β-cannabispirol, canniprene, cannabiripsol, and cannflavin B. Of particular interest was the high content of CBD observed in all preparations which could lead to a significant biological activity in synergism with other compounds. Tiago et al. [60] investigated deep eutectic solvents for the extraction of bioactive compounds from cannabis sativa L. flowers and/or leaves. The study found that cannabis leaves contain a variety of health-promoting compounds, including cannabigerol (CBG), quercetin, and kaempferol, which have anti-inflammatory, antioxidant, and neuroprotective effects. The study of Liu et al. [61] utilised 12 hemp leaf extracts from different germplasms and regions, specifically from Shanxi Province and Hunan Province, to investigate the content of CBD, THC, and CBN, shedding light on the potential therapeutic and industrial benefits of these compounds. Hemp leaves, especially those cultivated in Shanxi, reduced the release of pro-inflammatory cytokines and inhibited the cell morphological changes and membrane damage of lipopolysaccharide-induced inflammatory cells.

Cannabis leaves offer a multitude of benefits. By recognising the value of cannabis leaves and implementing efficient cultivation practices to minimise waste, we can maximise their potential as a green resource and harness their numerous advantages for human health-promoting, disease prevention, and environmental sustainability.

## 5. Conclusions

Cannabis leaves are known to contain a wide range of antioxidants, and this also includes cannabinoids, which have been shown to have significant health benefits. Using cannabis leaves as an antioxidant source can be a cost-effective option as they are often discarded during the cultivation of cannabis plants for their seeds or fibres. Moreover, the use of cannabis leaves as a source of antioxidants may also have environmental benefits as it can reduce waste and promote sustainable agriculture practices. This current study illuminates that not only the flowers and seeds of cannabis possess potential health benefits, but also the leaves; moreover, a comprehensive understanding is fostered regarding the diverse therapeutic potential inherent in this plant. The beneficial cannabinoid profile found in cannabis leaves was highlighted, as well as their antioxidant potential.

In this current research, the antioxidant potential of Białobrzeskie, Tygra, and Henola varieties obtained by ultrasound-assisted extraction and maceration by methanol, ethanol, isopropanol, and their 50:50 (*v*/*v*) mixtures were studied. All of the extracts have been found to possess antioxidant properties that can help protect against oxidative damage caused by free radicals. Białobrzeskie leaves extract obtained with ultrasound-assisted extraction with methanol was determined as the strongest antioxidant. Further research is needed to explore possible other directions of biological activity of cannabis leaves also involving in vivo studies. Other extraction techniques, various applications, and delivery methods might be suggested.

## Figures and Tables

**Table 1:** The content of cannabinoids present in Białobrzeskie, Henola, and Tygra leaves extracts obtained by ultrasound-assisted extraction (U-A.E), and maceration with the use of methanol (MOH), ethanol (EtOH), isopropanol (IOH), and 50:50 (*v*/*v*) mixtures of these solvents: CBD (cannabidiol), CBG (cannabigerol), Δ^9^-THC ((-)-delta 9-tetrahydrocannabinol), CBC (cannabichromene), as mg cannabinoid/g dry plant material.

Plant Material	Extraction	Extractant	CBD Content	CBG Content	Δ^9^-THC Content	CBC Content
			Mean Value ± SD			
			μg of Cannabinoid per Gram of Dry Plant Material (μg/g)			
Białobrzeskie L.	U-A.E	MOH	184.51 ± 5.61	6.10 ± 0.21	0.51 ± 0.01	0.71 ± 0.01
Białobrzeskie L.	U-A.E	EtOH	145.82 ± 4.32	6.04 ± 0.32	0.32 ± 0.02	0.74 ± 0.03
Białobrzeskie L.	U-A.E	IOH	145.83 ± 4.32	6.05 ± 0.22	0.32 ± 0.03	0.62 ± 0.05
Białobrzeskie L.	Maceration	MOH	182.71 ± 5.22	6.17 ± 0.32	0.43 ± 0.03	0.83 ± 0.05
Białobrzeskie L.	Maceration	EtOH	145.01 ± 0.91	5.57 ± 0.41	0.32 ± 0.02	0.71 ± 0.02
Białobrzeskie L.	Maceration	IOH	147.03 ± 2.60	5.64 ± 0.32	0.33 ± 0.02	0.72 ± 0.01
Białobrzeskie L.	U-A.E	MOH + EtOH	186.33 ± 4.13	6.35 ± 0.32	0.55 ± 0.02	0.82 ± 0.02
Białobrzeskie L.	U-A.E	EtOH + IOH	138.84 ± 2.53	5.66 ± 0.44	0.41 ± 0.01	0.73 ± 0.03
Białobrzeskie L.	U-A.E	MOH + IOH	137.74 ± 3.14	5.87 ± 0.75	0.32 ± 0.01	0.73 ± 0.02
Tygra L.	U-A.E	MOH	205.23 ± 3.11	8.44 ± 0.41	0.43 ± 0.02	0.84 ± 0.02
Tygra L.	U-A.E	EtOH	178.75 ± 7.41	8.55 ± 0.47	0.43 ± 0.02	0.75 ± 0.02
Tygra L.	U-A.E	IOH	159.13 ± 1.56	7.3 ± 0.35	0.34 ± 0.04	0.85 ± 0.03
Tygra L.	Maceration	MOH	201.13 ± 1.15	8.67 ± 0.61	0.41 ± 0.04	0.81 ± 0.04
Tygra L.	Maceration	EtOH	185.07 ± 7.14	8.64 ± 0.55	0.40 ± 0.05	0.84 ± 0.02
Tygra L.	Maceration	IOH	178.12 ± 2.46	8.11 ± 0.71	0.41 ± 0.04	0.83 ± 0.01
Tygra L.	U-A.E	MOH + EtOH	201.74 ± 5.01	8.32 ± 0.51	0.41 ± 0.04	0.82 ± 0.01
Tygra L.	U-A.E	EtOH + IOH	177.61 ± 7.41	8.04 ± 0.42	0.34 ± 0.03	0.72 ± 0.02
Tygra L.	U-A.E	MOH + IOH	181.30 ± 2.92	8.45 ± 0.32	0.43 ± 0.01	0.83 ± 0.01
Henola L.	U-A.E	MOH	173.41 ± 8.35	4.44 ± 0.33	0.13 ± 0.02	0.63 ± 0.01
Henola L.	U-A.E	EtOH	166.86 ± 6.23	5.12 ± 0.43	0.12 ± 0.01	0.62 ± 0.01
Henola L.	U-A.E	IOH	154.26 ± 4.21	4.40 ± 0.13	0.11 ± 0.01	0.64 ± 0.02
Henola L.	Maceration	MOH	210.91 ± 3.14	5.34 ± 0.34	0.11 ± 0.02	0.75 ± 0.03
Henola L.	Maceration	EtOH	155.32 ± 2.25	4.95 ± 0.41	0.12 ± 0.02	0.63 ± 0.03
Henola L.	Maceration	IOH	158.13 ± 1.63	4.64 ± 0.20	0.12 ± 0.03	0.64 ± 0.04
Henola L.	U-A.E	MOH + EtOH	195.64 ± 1.87	5.54 ± 0.22	0.13 ± 0.01	0.62 ± 0.04
Henola L.	U-A.E	EtOH + IOH	160.53 ± 4.63	5.02 ± 0.31	0.14 ± 0.02	0.60 ± 0.01
Henola L.	U-A.E	MOH + IOH	163.70 ± 5.88	5.21 ± 0.32	0.14 ± 0.04	0.61 ± 0.02

## Data Availability

Data are available in a publicly accessible repository.

## Supplementary Materials

The following supporting information can be downloaded at: https://www.mdpi.com/article/10.3390/antiox12071390/s1, Table S1: The results of the antioxidant activity of Białobrzeskie, Henola, and Tygra leaves extracts obtained by ultrasound-assisted extraction (U-A.E) with the use of methanol (MOH), ethanol (EtOH), isopropanol (IOH), presented as mg trolox/g plant material studied in DPPH, ABTS, CUPRAC, and FRAP assays. Means with different superscript letters (a, b, c) within the same column differ significantly (*p* < 0.05); Table S2: The results of the antioxidant activity of Białobrzeskie, Henola, and Tygra leaves extracts obtained by maceration with the use of methanol (MOH), ethanol (EtOH), isopropanol (IOH), presented as mg trolox/g plant material studied in DPPH, ABTS, CUPRAC, and FRAP assays. Means with different superscript letters (a–h) within the same column differ significantly (*p* < 0.05); Table S3: The results of the antioxidant activity of Białobrzeskie, Henola, and Tygra leaves extracts obtained by ultrasound-assisted extraction (U-A.E) with the use of 50:50 (*v*/*v*) mixtures of methanol (MOH), ethanol (EtOH), and isopropanol (IOH), presented as mg trolox/g plant material studied in DPPH, ABTS, CUPRAC, and FRAP assays. Means with different superscript letters (a–h) within the same column differ significantly (*p* < 0.05).

## References

[1] Karche T., Singh M. (2019). The Application of Hemp (*Cannabis Sativa* L.) for a Green Economy: A Review. Turk. J. Bot..

[2] Hesami M., Pepe M., Baiton A., Salami S.A., Jones A.M.P. (2022). New Insight into Ornamental Applications of Cannabis: Perspectives and Challenges. Plants.

[3] Krüger M., van Eeden T., Beswa D. (2022). *Cannabis Sativa* Cannabinoids as Functional Ingredients in Snack Foods—Historical and Developmental Aspects. Plants.

[4] Hesami M., Pepe M., Baiton A., Jones A.M.P. (2023). Current Status and Future Prospects in Cannabinoid Production through in Vitro Culture and Synthetic Biology. Biotechnol. Adv..

[5] Kovalchuk I., Pellino M., Rigault P., van Velzen R., Ebersbach J., Ashnest J.R., Mau M., Schranz M.E., Alcorn J., Laprairie R.B. (2020). The Genomics of Cannabis and Its Close Relatives. Annu. Rev. Plant Biol..

[6] Hesami M., Pepe M., Alizadeh M., Rakei A., Baiton A., Jones A.M.P. (2020). Recent Advances in Cannabis Biotechnology. Ind. Crops Prod..

[7] Zandkarimi F., Decatur J., Casali J., Gordon T., Skibola C., Nuckolls C. (2023). Comparison of the Cannabinoid and Terpene Profiles in Commercial Cannabis from Natural and Artificial Cultivation. Molecules.

[8] Chacon F.T., Raup-Konsavage W.M., Vrana K.E., Kellogg J.J. (2022). Secondary Terpenes in *Cannabis Sativa* L.: Synthesis and Synergy. Biomedicines.

[9] Thomas F.J., Kayser O. (2019). Minor Cannabinoids of *Cannabis Sativa* L.. J. Med. Sci..

[10] von Wrede R., Helmstaedter C., Surges R. (2021). Cannabidiol in the Treatment of Epilepsy. Clin. Drug Investig..

[11] Devinsky O., Cilio M.R., Cross H., Fernandez-Ruiz J., French J., Hill C., Katz R., Di Marzo V., Jutras-Aswad D., Notcutt W.G. (2014). Cannabidiol: Pharmacology and Potential Therapeutic Role in Epilepsy and Other Neuropsychiatric Disorders. Epilepsia.

[12] Vučković S., Srebro D., Vujović K.S., Vučetić Č., Prostran M. (2018). Cannabinoids and Pain: New Insights From Old Molecules. Front. Pharmacol..

[13] Jastrząb A., Jarocka-Karpowicz I., Skrzydlewska E. (2022). The Origin and Biomedical Relevance of Cannabigerol. Int. J. Mol. Sci..

[14] Izzo A.A., Capasso R., Aviello G., Borrelli F., Romano B., Piscitelli F., Gallo L., Capasso F., Orlando P., Di Marzo V. (2012). Inhibitory Effect of Cannabichromene, a Major Non-Psychotropic Cannabinoid Extracted from *Cannabis Sativa*, on Inflammation-Induced Hypermotility in Mice. Br. J. Pharmacol..

[15] Masyita A., Mustika Sari R., Dwi Astuti A., Yasir B., Rahma Rumata N., Emran T.B., Nainu F., Simal-Gandara J. (2022). Terpenes and Terpenoids as Main Bioactive Compounds of Essential Oils, Their Roles in Human Health and Potential Application as Natural Food Preservatives. Food Chem. X.

[16] Santana H.S.R., de Carvalho F.O., Silva E.R., Santos N.G.L., Shanmugam S., Santos D.N., Wisniewski J.O., Junior J.S.C., Nunes P.S., Araujo A.A.S. (2020). Anti-Inflammatory Activity of Limonene in the Prevention and Control of Injuries in the Respiratory System: A Systematic Review. Curr. Pharm. Des..

[17] Scandiffio R., Geddo F., Cottone E., Querio G., Antoniotti S., Gallo M.P., Maffei M.E., Bovolin P. (2020). Protective Effects of (E)-β-Caryophyllene (BCP) in Chronic Inflammation. Nutrients.

[18] Bautista J.L., Yu S., Tian L. (2021). Flavonoids in Cannabis Sativa: Biosynthesis, Bioactivities, and Biotechnology. ACS Omega.

[19] López-Lázaro M. (2009). Distribution and Biological Activities of the Flavonoid Luteolin. Mini Rev. Med. Chem..

[20] Batiha G.E.-S., Beshbishy A.M., Ikram M., Mulla Z.S., El-Hack M.E.A., Taha A.E., Algammal A.M., Elewa Y.H.A. (2020). The Pharmacological Activity, Biochemical Properties, and Pharmacokinetics of the Major Natural Polyphenolic Flavonoid: Quercetin. Foods.

[21] Tura M., Mandrioli M., Toschi T.G. (2019). Preliminary Study: Comparison of Antioxidant Activity of Cannabidiol (CBD) and α-Tocopherol Added to Refined Olive and Sunflower Oils. Molecules.

[22] Dawidowicz A.L., Typek R., Olszowy-Tomczyk M. (2023). Natural vs. Artificial Cannabinoid Oils: The Comparison of Their Antioxidant Activities. Eur. Food Res. Technol..

[23] Ekiner S.A., Gęgotek A., Skrzydlewska E. (2022). The Molecular Activity of Cannabidiol in the Regulation of Nrf2 System Interacting with NF-ΚB Pathway under Oxidative Stress. Redox Biol..

[24] Harvey B.S., Ohlsson K.S., Mååg J.L.V., Musgrave I.F., Smid S.D. (2012). Contrasting Protective Effects of Cannabinoids against Oxidative Stress and Amyloid-β Evoked Neurotoxicity in Vitro. NeuroToxicology.

[25] Biernacki M., Brzóska M.M., Markowska A., Gałażyn-Sidorczuk M., Cylwik B., Gęgotek A., Skrzydlewska E. (2021). Oxidative Stress and Its Consequences in the Blood of Rats Irradiated with UV: Protective Effect of Cannabidiol. Antioxidants.

[26] Pereira S.R., Hackett B., O’Driscoll D.N., Sun M.C., Downer E.J. (2021). Cannabidiol Modulation of Oxidative Stress and Signalling. Neuronal Signal.

[27] Booz G.W. (2011). Cannabidiol as an Emergent Therapeutic Strategy for Lessening the Impact of Inflammation on Oxidative Stress. Free Radic. Biol. Med..

[28] Kopustinskiene D.M., Masteikova R., Lazauskas R., Bernatoniene J. (2022). *Cannabis Sativa* L. Bioactive Compounds and Their Protective Role in Oxidative Stress and Inflammation. Antioxidants.

[29] Bhunia S., Kolishetti N., Arias A.Y., Vashist A., Nair M. (2022). Cannabidiol for Neurodegenerative Disorders: A Comprehensive Review. Front. Pharmacol..

[30] Cásedas G., Moliner C., Maggi F., Mazzara E., López V. (2022). Evaluation of Two Different *Cannabis Sativa* L. Extracts as Antioxidant and Neuroprotective Agents. Front. Pharmacol..

[31] Isidore E., Karim H., Ioannou I. (2021). Extraction of Phenolic Compounds and Terpenes from *Cannabis Sativa* L. By-Products: From Conventional to Intensified Processes. Antioxidants.

[32] Lazarjani M.P., Young O., Kebede L., Seyfoddin A. (2021). Processing and Extraction Methods of Medicinal Cannabis: A Narrative Review. J. Cannabis Res..

[33] AL Ubeed H.M.S., Bhuyan D.J., Alsherbiny M.A., Basu A., Vuong Q.V. (2022). A Comprehensive Review on the Techniques for Extraction of Bioactive Compounds from Medicinal Cannabis. Molecules.

[34] Pham-Huy L.A., He H., Pham-Huy C. (2008). Free Radicals, Antioxidants in Disease and Health. Int. J. Biomed. Sci..

[35] Liao H., Dong W., Shi X., Liu H., Yuan K. (2012). Analysis and Comparison of the Active Components and Antioxidant Activities of Extracts from *Abelmoschus esculentus* L.. Pharmacogn. Mag..

[36] Muzykiewicz A., Florkowska K., Nowak A., Zielonka-Brzezicka J., Klimowicz A. (2019). Antioxidant Activity of St. John’s Wort Extracts Obtained with Ultrasound-Assisted Extraction. Pomeranian J. Life Sci..

[37] Stasiłowicz A., Tykarska E., Lewandowska K., Kozak M., Miklaszewski A., Kobus-Cisowska J., Szymanowska D., Plech T., Jenczyk J., Cielecka-Piontek J. (2020). Hydroxypropyl-β-Cyclodextrin as an Effective Carrier of Curcumin-Piperine Nutraceutical System with Improved Enzyme Inhibition Properties. J. Enzym. Inhib. Med. Chem..

[38] Re R., Pellegrini N., Proteggente A., Pannala A., Yang M., Rice-Evans C. (1999). Antioxidant Activity Applying an Improved ABTS Radical Cation Decolorization Assay. Free Radic. Biol. Med..

[39] Stasiłowicz-Krzemień A., Rosiak N., Płazińska A., Płaziński W., Miklaszewski A., Tykarska E., Cielecka-Piontek J. (2022). Cyclodextrin Derivatives as Promising Solubilizers to Enhance the Biological Activity of Rosmarinic Acid. Pharmaceutics.

[40] Apak R., Güçlü K., Ozyürek M., Karademir S.E., Altun M. (2005). Total Antioxidant Capacity Assay of Human Serum Using Copper(II)-Neocuproine as Chromogenic Oxidant: The CUPRAC Method. Free Radic. Res..

[41] Keutgen A.J., Keutgen N., Wszelaczyńska E., Pobereżny J., Milczarek D., Tatarowska B., Flis B. (2020). Evaluation of Photosynthetic and Yield Traits in Ten Potato Clones and Cultivars Under Farming Conditions in Poland. Potato Res..

[42] Barska A., Jędrzejczak-Gas J., Wyrwa J. (2022). Poland on the Path towards Sustainable Development—A Multidimensional Comparative Analysis of the Socio-Economic Development of Polish Regions. Sustainability.

[43] Stasiłowicz A., Tomala A., Podolak I., Cielecka-Piontek J. (2021). *Cannabis Sativa* L. as a Natural Drug Meeting the Criteria of a Multitarget Approach to Treatment. Int. J. Mol. Sci..

[44] Kıran T.R., Otlu O., Karabulut A.B. (2023). Oxidative Stress and Antioxidants in Health and Disease. J. Lab. Med..

[45] Chaudhary P., Janmeda P., Docea A.O., Yeskaliyeva B., Razis A.F.A., Modu B., Calina D., Sharifi-Rad J. (2023). Oxidative Stress, Free Radicals and Antioxidants: Potential Crosstalk in the Pathophysiology of Human Diseases. Front. Chem..

[46] Martemucci G., Costagliola C., Mariano M., D’andrea L., Napolitano P., D’Alessandro A.G. (2022). Free Radical Properties, Source and Targets, Antioxidant Consumption and Health. Oxygen.

[47] Ferber S.G., Namdar D., Hen-Shoval D., Eger G., Koltai H., Shoval G., Shbiro L., Weller A. (2020). The “Entourage Effect”: Terpenes Coupled with Cannabinoids for the Treatment of Mood Disorders and Anxiety Disorders. Curr. Neuropharmacol..

[48] Atalay S., Jarocka-Karpowicz I., Skrzydlewska E. (2019). Antioxidative and Anti-Inflammatory Properties of Cannabidiol. Antioxidants.

[49] Jîtcă G., Ősz B.E., Vari C.E., Rusz C.-M., Tero-Vescan A., Pușcaș A. (2023). Cannabidiol: Bridge between Antioxidant Effect, Cellular Protection, and Cognitive and Physical Performance. Antioxidants.

[50] Mukhopadhyay P., Rajesh M., Horváth B., Bátkai S., Park O., Tanchian G., Gao R.Y., Patel V., Wink D.A., Liaudet L. (2011). Cannabidiol Protects against Hepatic Ischemia/Reperfusion Injury by Attenuating Inflammatory Signaling and Response, Oxidative/Nitrative Stress, and Cell Death. Free Radic. Biol. Med..

[51] di Giacomo V., Chiavaroli A., Recinella L., Orlando G., Cataldi A., Rapino M., Di Valerio V., Ronci M., Leone S., Brunetti L. (2020). Antioxidant and Neuroprotective Effects Induced by Cannabidiol and Cannabigerol in Rat CTX-TNA2 Astrocytes and Isolated Cortexes. Int. J. Mol. Sci..

[52] Kubiliene A., Mickute K., Baranauskaite J., Marksa M., Liekis A., Sadauskiene I. (2021). The Effects of *Cannabis Sativa* L. Extract on Oxidative Stress Markers In Vivo. Life.

[53] Surendran S., Qassadi F., Surendran G., Lilley D., Heinrich M. (2021). Myrcene—What Are the Potential Health Benefits of This Flavouring and Aroma Agent?. Front. Nutr..

[54] Salehi B., Upadhyay S., Orhan I.E., Jugran A.K., Jayaweera S.L.D., Dias A.D., Sharopov F., Taheri Y., Martins N., Baghalpour N. (2019). Therapeutic Potential of α- and β-Pinene: A Miracle Gift of Nature. Biomolecules.

[55] Tomko A.M., Whynot E.G., Dupré D.J. (2022). Anti-Cancer Properties of Cannflavin A and Potential Synergistic Effects with Gemcitabine, Cisplatin, and Cannabinoids in Bladder Cancer. J. Cannabis Res..

[56] Salehi B., Venditti A., Sharifi-Rad M., Kręgiel D., Sharifi-Rad J., Durazzo A., Lucarini M., Santini A., Souto E.B., Novellino E. (2019). The Therapeutic Potential of Apigenin. Int. J. Mol. Sci..

[57] Kubilienė A., Marksa M., Baranauskaitė J., Ragažinskienė O., Ivanauskas L. (2020). Comparative Evaluation of Antioxidant Activity of *Cannabis Sativa* L. Using FRAP and CUPRAP Assays. Chemija.

[58] Jin D., Dai K., Xie Z., Chen J. (2020). Secondary Metabolites Profiled in Cannabis Inflorescences, Leaves, Stem Barks, and Roots for Medicinal Purposes. Sci. Rep..

[59] De Vita S., Finamore C., Chini M.G., Saviano G., De Felice V., De Marino S., Lauro G., Casapullo A., Fantasma F., Trombetta F. (2022). Phytochemical Analysis of the Methanolic Extract and Essential Oil from Leaves of Industrial Hemp Futura 75 Cultivar: Isolation of a New Cannabinoid Derivative and Biological Profile Using Computational Approaches. Plants.

[60] Tiago F.J., Paiva A., Matias A.A., Duarte A.R.C. (2022). Extraction of Bioactive Compounds From *Cannabis Sativa* L. Flowers and/or Leaves Using Deep Eutectic Solvents. Front. Nutr..

[61] Liu Y., Xiao A.-P., Cheng H., Liu L.-L., Kong K.W., Liu H.-Y., Wu D.-T., Li H.-B., Gan R.-Y. (2022). Phytochemical Differences of Hemp (*Cannabis Sativa* L.) Leaves from Different Germplasms and Their Regulatory Effects on Lipopolysaccharide-Induced Inflammation in Matin-Darby Canine Kidney Cell Lines. Front. Nutr..

