# Supplementary material for: Determining Antioxidant Activity of Cannabis Leaves Extracts from Different Varieties—Unveiling Nature’s Treasure Trove
Source: Antioxidants (Basel). 2023 Jul 6;12(7):1390. doi: 10.3390/antiox12071390 (PMC10376652; doi:10.3390/antiox12071390)
Supplement: Supplementary file 1 [file antioxidants-12-01390-s001.zip › antioxidants-2479785-supplementary.pdf]

Supplementary materials

Table S1. The results of the antioxidant activity of Białobrzeskie, Henola, and Tygra leaves extracts obtained by ultrasound-assisted extraction (U-A.E) with the use of methanol (MOH), ethanol (EtOH), isopropanol (IOH), presented as mg trolox/g plant material studied in DPPH, ABTS, CUPRAC, and FRAP assays. Means with different superscript letters (a–h) within the same column differ significantly ($p < 0.05$)

Plant material	Extraction	Extractant	DPPH	ABTS	CUPRAC	FRAP
			Mean ± SD (mg trolox/ g plant material)			
Białobrzeskie L.	U-A.E	MOH	10.288 ± 0.103^{g}	9.572 ± 0.029^{g}	22.195 ± 0.242^{h}	11.066 ± 0.048^{f}
Białobrzeskie L.	U-A.E	EtOH	7.932 ± 0.050^{f}	8.574 ± 0.008^{d}	11.796 ± 0.279^{d}	6.925 ± 0.046^{d}
Białobrzeskie L.	U-A.E	IOH	7.782 ± 0.015^{f}	7.837 ± 0.029^{c}	6.528 ± 0.123^{b}	3.690 ± 0.022^{b}
Tygra L.	U-A.E	MOH	6.955 ± 0.023^{e}	10.368 ± 0.035^{h}	16.008 ± 0.140^{g}	10.287 ± 0.148^{e}
Tygra L.	U-A.E	EtOH	4.248 ± 0.033^{d}	9.176 ± 0.017^{f}	13.633 ± 0.014^{f}	6.607 ± 0.072^{d}
Tygra L.	U-A.E	IOH	2.851 ± 0.027^{b}	8.608 ± 0.042^{d}	7.706 ± 0.120^{c}	4.059 ± 0.015^{c}
Henola L.	U-A.E	MOH	4.019 ± 0.057^{c}	8.839 ± 0.030^{e}	12.789 ± 0.049^{e}	6.375 ± 0.036^{d}
Henola L.	U-A.E	EtOH	2.839 ± 0.021^{b}	7.522 ± 0.013^{b}	7.333 ± 0.004^{c}	4.028 ± 0.032^{c}
Henola L.	U-A.E	IOH	2.198 ± 0.004^{a}	7.202 ± 0.020^{a}	5.878 ± 0.058^{a}	3.060 ± 0.028^{a}

Table S2. The results of the antioxidant activity of Białobrzeskie, Henola, and Tygra leaves extracts obtained by maceration with the use of methanol (MOH), ethanol (EtOH), isopropanol (IOH), presented as mg trolox/g plant material studied in DPPH, ABTS, CUPRAC, and FRAP assays. Means with different superscript letters (a–h) within the same column differ significantly ($p < 0.05$)

Plant material	Extraction	Extractant	DPPH	ABTS	CUPRAC	FRAP
			Mean ± SD (mg trolox/ g plant material)			
Białobrzeskie L.	Maceration	MOH	5.632 ± 0.046^{g}	10.239 ± 0.105^{f}	11.573 ± 0.147^{e}	11.066 ± 0.048^{g}
Białobrzeskie L.	Maceration	EtOH	2.993 ± 0.027^{b}	9.164 ± 0.048c	8.698 ± 0.155^{d}	6.925 ± 0.046^{e}
Białobrzeskie L.	Maceration	IOH	4.104 ± 0.040^{e}	9.157 ± 0.022^{c}	13.729 ± 0.114^{f}	3.690 ± 0.022^{a}
Tygra L.	Maceration	MOH	4.685 ± 0.092^{f}	10.160 ± 0.017^{f}	14.392 ± 0.084^{g}	10.287 ± 0.148^{f}
Tygra L.	Maceration	EtOH	3.340 ± 0.059^{c}	9.526 ± 0.050^{d}	7.683 ± 0.116^{c}	6.607 ± 0.072^{d}
Tygra L.	Maceration	IOH	3.789 ± 0.010^{d}	9.880 ± 0.031^{e}	15.766 ± 0.091^{h}	4.059 ± 0.015^{b}
Henola L.	Maceration	MOH	4.566 ± 0.040^{f}	8.662 ± 0.057^{b}	7.444 ± 0.137^{c}	4.934 ± 0.021^{c}
Henola L.	Maceration	EtOH	2.183 ± 0.016^{a}	8.082 ± 0.045^{a}	4.140 ± 0.147^{a}	3.486 ± 0.011^{a}
Henola L.	Maceration	IOH	2.867 ± 0.019^{b}	8.089 ± 0.083^{a}	5.628 ± 0.174^{b}	4.737 ± 0.008^{c}

Table S3. The results of the antioxidant activity of Białobrzeskie, Henola, and Tygra leaves extracts obtained by ultrasound-assisted extraction (U-A.E) with the use of 50:50 (v/v) mixtures of methanol (MOH), ethanol (EtOH), and isopropanol (IOH), presented as mg trolox/g plant material studied in DPPH, ABTS, CUPRAC, and FRAP assays. Means with different superscript letters (a–h) within the same column differ significantly ($p < 0.05$)

Plant material	Extraction	Extractant	DPPH	ABTS	CUPRAC	FRAP
			Mean ± SD (mg trolox/ g plant material)			
Białobrzeskie L.	U-A.E	MOH + EtOH	7.563 ± 0.075^{h}	8.130 ± 0.097^{d}	15.992 ± 0.024^{h}	10.250 ± 0.135^{h}
Białobrzeskie L.	U-A.E	EtOH + IOH	5.600 ± 0.075^{f}	8.303 ± 0.097^{d,e}	12.437 ± 0.037^{g}	8.012 ± 0.040^{f}
Białobrzeskie L.	U-A.E	MOH + IOH	2.815 ± 0.033^{c}	7.564 ± 0.079^{c}	6.205 ± 0.055^{b}	3.693 ± 0.031^{c}
Tygra L.	U-A.E	MOH + EtOH	6.141 ± 0.112^{g}	8.481 ± 0.116^{e}	10.235 ± 0.012^{e}	8.942 ± 0.104^{g}
Tygra L.	U-A.E	EtOH + IOH	4.347 ± 0.084^{d}	8.169 ± 0.025^{d}	11.789 ± 0.039^{f}	5.900 ± 0.035^{e}
Tygra L.	U-A.E	MOH + IOH	2.526 ± 0.019^{b}	7.611 ± 0.037^{c}	6.723 ± 0.028^{d}	3.172 ± 0.017^{b}
Henola L.	U-A.E	MOH + EtOH	4.681 ± 0.055^{e}	6.832 ± 0.120^{b}	6.553 ± 0.038^{c,d}	4.165 ± 0.094^{d}
Henola L.	U-A.E	EtOH + IOH	3.034 ± 0.043^{c}	6.983 ± 0.011^{b}	6.453 ± 0.173^{b,c}	4.223 ± 0.041^{d}
Henola L.	U-A.E	MOH + IOH	2.013 ± 0.051^{a}	6.502 ± 0.029^{a}	5.008 ± 0.062^{a}	2.272 ± 0.021^{a}
